# Primary hyperparathyroidism as first manifestation in multiple endocrine neoplasia type 2A: an international multicenter study

**DOI:** 10.1530/EC-20-0163

**Published:** 2020-05-06

**Authors:** Louise Vølund Larsen, Delphine Mirebeau-Prunier, Tsuneo Imai, Cristina Alvarez-Escola, Kornelia Hasse-Lazar, Simona Censi, Luciana A Castroneves, Akihiro Sakurai, Minoru Kihara, Kiyomi Horiuchi, Véronique Dorine Barbu, Francoise Borson-Chazot, Anne-Paule Gimenez-Roqueplo, Pascal Pigny, Stephane Pinson, Nelson Wohllk, Charis Eng, Berna Imge Aydogan, Dhananjaya Saranath, Sarka Dvorakova, Frederic Castinetti, Attila Patocs, Damijan Bergant, Thera P Links, Mariola Peczkowska, Ana O Hoff, Caterina Mian, Trisha Dwight, Barbara Jarzab, Hartmut P H Neumann, Mercedes Robledo, Shinya Uchino, Anne Barlier, Christian Godballe, Jes Sloth Mathiesen

**Affiliations:** 1 Department of ORL Head & Neck Surgery and Audiology, Odense University Hospital, Odense, Denmark; 2 Laboratoire de Biochimie et Biologie Moléculaire, CHU Angers, Université d’Angers, UMR CNRS 6015, INSERM U1083, MITOVASC, Angers, France; 3 Department of Breast & Endocrine Surgery, National Hospital Organization, Higashinagoya National Hospital, Nagoya, Japan; 4 Endocrinology and Nutrition Department, University Hospital ‘La Paz’, Madrid, Spain; 5 Department of Nuclear Medicine and Endocrine Oncology, Maria Sklodowska-Curie National Research Institute of Oncology, Gliwice Branch, Gliwice, Poland; 6 Endocrinology Unit, Department of Medicine (DIMED), University of Padua, Padua, Italy; 7 Department of Endocrinology, Endocrine Oncology Unit, Instituto do Cancer do Estado de São Paulo, Faculdade de Medicina da Universidade de São Paulo, São Paulo, Brazil; 8 Department of Medical Genetics and Genomics, Sapporo Medical University School of Medicine, Sapporo, Japan; 9 Department of Surgery, Kuma Hospital, Kobe, Hyogo, Japan; 10 Department of Breast and Endocrine Surgery, Tokyo Women’s Medical University, Tokyo, Japan; 11 AP-HP, Sorbonne Université, Laboratoire Commun de Biologie et Génétique Moléculaires, Hôpital St Antoine & INSERM CRSA, Paris, France; 12 Réseau TenGen, Marseille, France; 13 Fédération d’Endocrinologie, Hospices Civils de Lyon, Université Lyon 1, France; 14 Service de Génétique, AP-HP, Hôpital européen Georges Pompidou, Paris, France; 15 Université de Paris, PARCC, INSERM, Paris, France; 16 Laboratoire de Biochimie et Oncologie Moléculaire, CHU Lille, Lille, France; 17 Laboratoire de Génétique Moléculaire, CHU Lyon, Lyon, France; 18 Endocrine Section, Hospital del Salvador, Santiago de Chile, Department of Medicine, University of Chile, Santiago, Chile; 19 Genomic Medicine Institute, Lerner Research Institute and Taussig Cancer Institute, Cleveland Clinic, Cleveland, Ohio, USA; 20 Department of Endocrinology And Metabolic Diseases, Ankara University School of Medicine, Ankara, Turkey; 21 Department of Research Studies & Additional Projects, Cancer Patients Aid Association, Dr. Vithaldas Parmar Research & Medical Centre, Worli, Mumbai, India; 22 Department of Molecular Endocrinology, Institute of Endocrinology, Prague, Czech Republic; 23 Aix-Marseille Université, Institut National de la Santé et de la Recherche Médicale (INSERM), U1251, Marseille Medical Genetics (MMG), Marseille, France; 24 Department of Endocrinology, Assistance Publique-Hôpitaux de Marseille (AP-HM), Hôpital de la Conception, Centre de Référence des Maladies Rares de l’hypophyse HYPO, Marseille, France; 25 HAS-SE Momentum Hereditary Endocrine Tumors Research Group, Semmelweis University, Budapest, Hungary; 26 Department of Surgical Oncology, Institute of Oncology, Ljubljana, Slovenia; 27 Department of Endocrinology, University of Groningen, University Medical Center Groningen, Groningen, Netherlands; 28 Department of Hypertension, Institute of Cardiology, Warsaw, Poland; 29 Cancer Genetics, Kolling Institute, Royal North Shore Hospital and University of Sydney, Sydney, New South Wales, Australia; 30 Department of Nuclear Medicine and Endocrine Oncology, Maria Sklodowska-Curie National Research Institute of Oncology, Gliwice Branch, Gliwice, Poland; 31 Section for Preventive Medicine, Medical Center-University of Freiburg, Faculty of Medicine, Albert Ludwigs-University of Freiburg, Freiburg, Germany; 32 Hereditary Endocrine Cancer Group, Spanish National Cancer Research Center (CNIO), Madrid, Spain; 33 Centro de Investigación Biomédica en Red de Enfermedades Raras (CIBERER), Madrid, Spain; 34 Department of Endocrine Surgery, Noguchi Thyroid Clinic and Hospital Foundation, Beppu, Oita, Japan; 35 Aix Marseille Univ, APHM, INSERM, MMG, Laboratory of Molecular Biology, Hospital La Conception, Marseille, France; 36 Department of Clinical Research, University of Southern Denmark, Odense, Denmark

**Keywords:** primary hyperparathyroidism, multiple endocrine neoplasia type 2A, *RET*, medullary thyroid carcinoma, pheochromocytoma

## Abstract

**Objective:**

Multiple endocrine neoplasia type 2A (MEN 2A) is a rare syndrome caused by *RET* germline mutations and has been associated with primary hyperparathyroidism (PHPT) in up to 30% of cases. Recommendations on *RET* screening in patients with apparently sporadic PHPT are unclear. We aimed to estimate the prevalence of cases presenting with PHPT as first manifestation among MEN 2A index cases and to characterize the former cases.

**Design and methods:**

An international retrospective multicenter study of 1085 MEN 2A index cases. Experts from MEN 2 centers all over the world were invited to participate. A total of 19 centers in 17 different countries provided registry data of index cases followed from 1974 to 2017.

**Results:**

Ten cases presented with PHPT as their first manifestation of MEN 2A, yielding a prevalence of 0.9% (95% CI: 0.4–1.6). 9/10 cases were diagnosed with medullary thyroid carcinoma (MTC) in relation to parathyroid surgery and 1/10 was diagnosed 15 years after parathyroid surgery. 7/9 cases with full TNM data were node-positive at MTC diagnosis.

**Conclusions:**

Our data suggest that the prevalence of MEN 2A index cases that present with PHPT as their first manifestation is very low. The majority of index cases presenting with PHPT as first manifestation have synchronous MTC and are often node-positive. Thus, our observations suggest that not performing *RET* mutation analysis in patients with apparently sporadic PHPT would result in an extremely low false-negative rate, if no other MEN 2A component, specifically MTC, are found during work-up or resection of PHPT.

## Introduction

Multiple endocrine neoplasia type 2 (MEN 2) is an autosomal dominant inherited cancer syndrome caused by germline mutations of the rearranged during transfection (*RET*) proto-oncogene (1, 2, 3, 4, 5, 6). The syndrome is divided into MEN 2A and MEN 2B with a point prevalence of 13–24 per million and 1–2 per million, respectively (7, 8, 9, 10). Virtually all patients with MEN 2A develop medullary thyroid carcinoma (MTC), while lower numbers develop pheochromocytoma, primary hyperparathyroidism (PHPT), cutaneous lichen amyloidosis (CLA) and Hirschsprung disease (HSCR) (11).

For identification of new MEN 2A index cases and families, *RET* screening has been recommended for years in all patients with apparently sporadic MTC, pheochromocytoma, CLA and infants with HSCR (11, 12, 13, 14). However, for patients with apparently sporadic PHPT, recommendations on *RET* screening are less clear. Thus, in 2001 the consensus guidelines from the seventh international workshop on MEN recommended against *RET* screening in these patients (13), while the issue lacks mentioning in the 2009 and 2015 guidelines by the American Thyroid Association (11, 12).

To ascertain if all patients with apparently sporadic PHPT should be *RET* screened, a valuable estimate would be the prevalence of MEN 2A index cases presenting with PHPT as first manifestation in an unselected population-based cohort of apparently sporadic PHPT cases, who have all been *RET* screened. To our knowledge, however, no such cohorts exist. Instead, a surrogate cohort study is to examine the prevalence of MEN 2A index cases presenting with PHPT as the first manifestation in an unselected cohort of MEN 2A index cases. Based on the experience from previous MEN 2A PHPT series (15, 16), we hypothesized that this prevalence would be low.

Consequently, we aimed to estimate the prevalence of MEN 2A index cases presenting with PHPT as first manifestation in an unselected cohort of MEN 2A index cases. Additionally, we aimed to characterize the cases presenting with PHPT as their first manifestation.

## Methods

### Study design and participants

This investigation is an international retrospective multicenter study of 1085 MEN 2A index cases. We invited experts from 40 MEN 2 centers all over the world to participate. This yielded a total of 19 centers in 17 different countries, including Denmark, providing data of index cases followed from 1974 to 2017 (Supplementary Table 1, see section on supplementary materials given at the end of this article). Data were retrieved from June 2017 to September 2019.

### Data sources

Data were drawn from the registry of each center. Some of the patients have been reported on previous occasions and updated data were obtained (17, 18, 19, 20, 21, 22, 23, 24, 25, 26).

### Variables

Patients were defined as having MEN 2 if they had tested positive for a *RET* germline sequence change classified as pathogenic (mutation) in the ARUP MEN 2 database on February 1, 2020 (27). For inclusion of only the MEN 2A patients, we excluded those with mutations pathognomonic of MEN 2B (*RET* M918T and A883F) (28, 29). An index case was defined as a clinically affected individual through whom attention is first drawn to MEN 2A in a family (https://www.cancer.gov/publications/dictionaries/genetics-dictionary/def/index-case). The first manifestation in MEN 2A was defined by the symptoms or biochemistry leading to initial endocrine work-up and was judged by the MEN 2 experts participating in the study. PHPT had to be both biochemically (hypercalcemia and an elevated or inappropriately normal parathyroid hormone level (30)) and histologically proven, while MTC, pheochromocytoma, CLA and HSCR were considered by histology only. TNM staging was performed according to the seventh edition of the American Joint Committee on Cancer Staging Manual (31). Biochemical cure was regarded as undetectable basal calcitonin at last biochemical follow-up.

### Statistical analysis

Continuous data were presented as median and range. All analyses were done using Stata® 15.1 (StataCorp LP).

### Ethics

Informed consent was given by all patients participating in the study for *RET* screening. Ethical approval was obtained from the institutional review boards of all participating centers when required: French National Commission for Computerized Data and Individual Freedom, Institutional Ethical Review Board of Shinshu University School of Medicine (Matsumoto, Japan), Comité de bioética y bienestar animal of the Instituto de Salud Carlos III, Northern Sydney Local Health District Human Research Ethics Committee, ICESP/HCFMUSP, Ethics Committee of the Institute of Cardiology (Warsaw, Poland), Regional Committee on Health Research Ethics for Southern Denmark, Scientific and Research Committee of the Medical Research Council of Hungary, Ethics Committee of Aix Marseille University, Ethics Committee of the Institute of Endocrinology (Prague, Czech Republic), Ethics Committee of Reliance Life Sciences (Navi Mumbai, India), Local Ethics Committee of Ankara University Faculty of Medicine, Cleveland Clinic Institutional Review Board for Human Subjects Protection and Ethical Committee (Santiago, Chile). This was in accordance with the ethical standards of each country and center.

The investigation was approved by the respective institutional review boards for human subjects protection in accordance with the ethical standards of each country and center.

## Results

A total of 1085 MEN 2A index cases were included in the study. The distribution of *RET* germline mutations in these cases is shown in Table 1. The most frequent site of mutations was exon 11 (53%), followed by exon 10 (25%), exon 14 (12%), exon 13 (7%), exon 15 (3%), exon 8 (1%) and exon 16 (0%). Of the 1085 cases, 10 had presented with PHPT as first manifestation of the syndrome, yielding a prevalence of 0.9% (95% CI: 0.4–1.6).

**Table 1:** Distributions of *RET* mutations among 1085 MEN 2A index cases.

*RET* mutation	*n*	(%)
Exon 8		
C531R	3	(0)
G533C	5	(0)
G548S	2	(0)
Exon 10		
C609F/G/R/S/Y	19	(2)
C611F/G/W/Y	48	(4)
C618F/G/R/S/W/Y	113	(10)
C620F/G/R/S/W/Y	87	(8)
Exon 11		
C630R/Y	4	(0)
D631Y	3	(0)
C634F/G/L/S/R/W/Y	562	(52)
K666E/N/T	6	(1)
Exon 13		
E768D	18	(2)
Q781R	1	(0)
L790F	52	(5)
Exon 14		
V804L/M	132	(12)
Exon 15		
S891A	28	(3)
Exon 16		
R912P	1	(0)
M918V	1	(0)
Total	1085	(100)

Due to rounding up, not all sums of the numbers fit.

MEN 2A, multiple endocrine neoplasia type 2A; *RET*, rearranged during transfection.

Characteristics of the ten cases are depicted in Table 2. In these cases, the female-to-male ratio was 4.0 (95% CI: −2.2–10.2), while the median age at diagnosis of PHPT was 34.5 years (range, 14–68). All cases were diagnosed with PHPT between 1993 and 2012. Of these, seven were diagnosed in the new millennium.

**Table 2:** Characteristics of MEN 2A index cases presenting with PHPT as first manifestation.

Patient no.	Sex	*RET* mutation	PHPT^a^			MTC^b^		PHEO^b^		HSCR^b^	CLA^b^	Follow-up	
			Age (yrs)	Histology	Symptoms	Age (yrs)	TNM^c^	Age (yrs)	Side			Age (yrs)	Status
1	F	C634Y	14	Hyperplasia	Y	14	T2N1M0		None	N	N	19	Alive
2	F	C634R	18	Adenoma	Y	18	T2N1M0	18	Bilateral^d^	N	N	30	Dead
3	M	C634Y	19	Adenoma	Y	19	T2N0M0	27	Unilateral	N	N	30	Alive
4	F	C634R	28	Hyperplasia	Y	28	T1N1M0	28	Unilateral	N	N	38	Alive
5	F	C634R	31	Adenoma	Y	46	T1N0N0	42	Bilateral	N	N	57	Alive
6	F	C634R	38	Hyperplasia	Y	38	T2N1M0	38	Bilateral	N	N	47	Alive
7	F	C611Y	40	Adenoma	Y	40	T1N1M0	40	Unilateral	N	N	47	Alive
8	M	C620R	61	Adenoma	Y	61	T3N1M1		None	N	N	75	Dead
9	F	E768D	61	Adenoma	Y	61	T1N1M0		None	N	N	66	Alive
10	F	C618F	68	Adenoma	NA	68	T2NxMx	80	Unilateral	N	N	90	Alive

^a^Defined by biochemistry (30) and histology. ^b^Defined by histology. ^c^Staging was based on the American Joint Committee on Cancer seventh edition (31). ^d^Malignant.

CLA, cutaneous lichen amyloidosis; HSCR, Hirschsprung disease; MEN 2A, multiple endocrine neoplasia type 2A; MTC, medullary thyroid carcinoma; N, no; NA, not available; PHEO, pheochromocytoma; PHPT, primary hyperparathyroidism; RET, rearranged during transfection; Y, yes.

All cases with pertinent data (*n* = 9) were symptomatic at diagnosis of PHPT with symptoms being nephrolithiasis (*n* = 8) and polyuria (*n* = 1). MTC was diagnosed in 10/10 cases. 9/10 were diagnosed in relation to parathyroid surgery as a synchronous MTC and 1/10 was diagnosed 15 years after parathyroid surgery, as a metachronous MTC. In three cases, MTC was not suspected during preoperative PHPT work-up, but diagnosed during parathyroid surgery. 7/9 cases with full TNM data available had regional lymph node metastases at time of MTC diagnosis. Biochemical cure was achieved only in the node-negative cases (*n* = 2).

## Discussion

This large international retrospective multicenter study found that 0.9% of cases had PHPT as their first manifestation of MEN 2A. In the cases presenting with PHPT as first manifestation, MTC was coexistent and had metastasized to regional lymph nodes in 7/9 cases.

### Prevalence

In this study, we found 0.9% of our MEN 2A index cases presented with PHPT as the first manifestation of the syndrome. To our knowledge, no similar studies on MEN 2A index cases have been reported, rendering comparisons difficult. However, there exist several studies, in which the study cohorts comprise only MEN 2A cases with PHPT. In these cohorts the prevalence of MEN 2A cases presenting with PHPT as a first manifestation ranges 0–11% (15, 16, 32, 33, 34, 35). Considering the selection of these cohorts and the fact that they included index and non-index cases, presumably a majority of the latter, our prevalence of 0.9% appears as a solid estimate. This is in line with the experience of other smaller series, that PHPT rarely was the first diagnosed manifestation (16, 36). In fact, there seems to be a decrease in the overall prevalence of PHPT in MEN 2A cohorts reported over time, possibly explained by inclusion of more patients with the full-blown syndrome (MTC, pheochromocytoma and PHPT) in the earliest series (6, 33, 37).

In our overall cohort, the most frequently mutated codon was 634, followed by codons 804, 618, 620, 790, 611, 891, 609, 768 and other rarely mutated codons. With only minor differences, likely accounted for by founder effects, the distribution of mutations in our cohort is, by and large, comparable to that of series in the literature (7, 17, 19, 20, 21, 38, 39, 40, 41, 42, 43, 44, 45).

### Characteristics of cases

Our study depicts the characteristics of MEN 2A index cases presenting with PHPT as first manifestation. Age at diagnosis is by and large similar to that of other MEN 2A PHPT cohorts (15, 16, 32, 33, 35, 46). Our female-to-male ratio of 4.0 is higher than that (1.3–1.9) reported by others (15, 16, 32, 34). This may be a question of sample size, but may also indicate that female MEN 2A cases in comparison to males are more prone to present with PHPT as first manifestation.

In our cohort all cases with pertinent data were symptomatic at diagnosis of PHPT. This is in contrast with other MEN 2A PHPT cohorts, in which most cases (58–84%) are asymptomatic (15, 16, 32, 33, 34). A likely explanation is the difference in cohorts, where our cohort solely comprises index cases presenting with PHPT as first manifestation, while the other cohorts presumably comprise mainly non-index cases diagnosed with PHPT by screening before they become symptomatic.

Nine of our ten cases were diagnosed with MTC, either due to a suspected or unsuspected finding in relation to parathyroid surgery. As a consequence, *RET* screening would be prompted by the MTC, if not instigated by the PHPT diagnosis. To our knowledge, the MTC TNM stage of the cases has not previously been reported in MEN 2A PHPT cohorts. In our cohort, 7/9 cases with available data were MTC node positive. This may reflect an over-representation of codon 634 mutation carriers (6/10), who generally have earlier age at MTC onset compared with other MEN 2A patients (47, 48). The over-representation in this cohort of MEN2A index cases presenting with PHPT as first manifestation is expected, as carriers of codon 634 mutations are regarded as having the highest penetrance of PHPT (6, 46). Given the fact, that long-term biochemical cure only rarely occurs in node-positive MTC (49), the likelihood of cure as indicated by our cohort is supposedly very low for MEN 2A index cases that present with PHPT as their first manifestation. Due to the high prevalence of regional lymph node metastases in these cases, neck dissection is often warranted already at primary surgery for better local control. Although controversial, the preoperative serum calcitonin level may also guide this decision, despite the fact that high levels not always guarantee metastases (50, 51, 52). On a general comment, the cohort of cases presenting with PHPT as first manifestation is small making generalizations difficult.

### Limitations

To assess if all cases with apparently sporadic PHPT should be *RET* screened, one could have estimated the prevalence of MEN 2A index cases presenting with PHPT as first manifestation in an unselected population-based cohort of cases with apparently sporadic PHPT, in which all had been *RET* screened. To our knowledge, no such cohorts exist, rendering such a study unfeasible. Instead, we sought to estimate the prevalence of MEN 2A index cases presenting with PHPT as their first manifestation in the largest series of MEN 2A index cases seen to date.

An issue that may underestimate the prevalence is the fact that our study cohort consists of already recognized MEN 2A index cases. Thus, we cannot rule out that some MEN 2A index cases presenting with PHPT as first manifestation, are still unrecognized as MEN 2A cases, if they have not been *RET* screened and instead are still regarded as sporadic PHPT cases. To comply with this, a study cohort of apparently sporadic PHPT cases is needed as previously described. However, as the first *RET* germline mutations causing MEN 2A were discovered >25 years ago (1, 2) combined with the fact that *de novo* mutations rarely occur (53), one may argue that the pool of unrecognized MEN 2A families arising from de novo mutations likely is very small, thus minimizing the issue.

As in several other multicenter studies on MEN 2, selection bias in the current study cannot be ruled out (6, 15, 28, 29, 54, 55, 56, 57, 58, 59, 60, 61, 62, 63). Including all MEN 2 centers in the world is an immensely difficult and time-consuming task. However, formation of a consortium including all MEN 2 centers worldwide may be helpful for future studies.

A limitation of the study is the lack of preoperative data, especially regarding ultrasonography and serum calcitonin. This hinders the elaborations on reasons for the preoperative suspicion of MTC during PHPT work-up and makes it difficult to assess potential diagnostic bias. High-resolution ultrasonography is routinely used in the preoperative setup for PHPT patients, while measurements of serum calcitonin are not (64). In some patients the preoperative serum calcitonin will likely be measured as a consequence of thyroid nodules found by ultrasonography (65, 66, 67, 68, 69). Some authors have suggested systematically preoperative calcitonin measurements in patients with apparently sporadic PHPT to exclude potential MEN 2 cases (70). Such a strategy in all PHPT patients or in PHPT patients with synchronous thyroid tumors found by ultrasonography would likely prove more cost effective than systematically carrying out *RET* mutation analysis. However, to our knowledge no evidence for or against this strategy exists.

## Conclusion

Our data suggest that the prevalence of MEN 2A index cases that present with PHPT as their first manifestation is very low. The majority of index cases presenting with PHPT as first manifestation, have synchronous MTC, often node-positive. Thus, our observations suggest that not performing *RET* mutation analysis in patients with apparently sporadic PHPT would result in an extremely low false negative rate, if no other MEN 2A component, specifically MTC, are found during work-up or resection of PHPT.

## Supplementary Material

Appendix 1. MEN 2A index cases.

## Declaration of interest

The authors declare that there is no conflict of interest that could be perceived as prejudicing the impartiality of the research reported.

## Funding

S D received a national grant (AZV 16-32665A).

## Author contribution statement

J S Mathiesen conceived the study, drafted, revised and approved the manuscript. L V Larsen collected the data, revised and approved the manuscript. The remaining authors contributed data, critically revised and gave final approval of the manuscript.
