# Supplementary material for: Primary hyperparathyroidism as first manifestation in multiple endocrine neoplasia type 2A: an international multicenter study
Source: Endocr Connect. 2020 May 6;9(6):489–97. doi: 10.1530/EC-20-0163 (PMC7354718; doi:10.1530/EC-20-0163)
Supplement: Appendix 1. MEN 2A index cases. [file supplementary_table_1.pdf]

Appendix 1. MEN 2A index cases.

Center	Country	Principal investigator	Patients
TenGen	France	Delphine Prunier-Mirebeau	356
Matsumoto	Japan	Akihiro Sakurai	229
Madrid	Spain	Mercedes Robledo	80
Freiburg	Germany	Hartmut P. H. Neumann	75
Gliwice	Poland	Barbara Jarzab	57
Sydney	Australia	Trisha Dwight	40
Padua	Italy	Caterina Mian	36
Sao Paulo	Brazil	Ana O. Hoff	32
Warsaw	Poland	Mariola Peczkowska	28
Denmark	Denmark	Jes Sloth Mathiesen	28
Gronningen	Netherlands	Thera P. Links	23
Ljubljana	Slovenia	Damijan Bergant	22
Budapest	Hungary	Attila Patocs	16
Marseille	France	Frederic Castinetti	16
Prague	Czech Republic	Sarka Dvorakova	16
Mumbai	India	Dhananjaya Saranath	13
Ankara	Turkey	Berna Imge Aydogan	10
Cleveland	US	Charis Eng	5
Santiago de Chile	Chile	Nelson Wohllk	3

Abbreviations: MEN 2A, multiple endocrine neoplasia 2A.
